# Assessment of Neutron Radiation Effects on the Fiber Optics Current Sensor Performance During JET DTE2 Experimental Campaign

**DOI:** 10.3390/s25216552

**Published:** 2025-10-24

**Authors:** Andrei Gusarov, Perry Beaumont, JET Contributors

**Affiliations:** 1 SCK CEN, 2400 Mol, Belgium; 2 United Kingdom Atomic Energy Authority, Culham Campus, Abingdon OX14 3DB, Oxfordshire, UK

**Keywords:** fibre optics current sensor (FOCS), JET, ITER, DTE2, nuclear radiation

## Abstract

Fibre Optics Current Sensor (FOCS) will be used at ITER to perform plasma current measurement during quasi-steady state D-T plasma operation. Effects of the tokamak harsh environment on the FOCS performance must be evaluated to predict possible failure modes and relevant mitigation measures. The influence of nuclear radiation with the significant flux of 14 MeV neutrons is of specific concern. This problem was addressed by operating the FOCS during D-T campaign at JET (DTE2). In the present report experimental results are presented and analysed. These results indicate that FOCS will effectively perform current measurements during ITER nuclear operation.

## 1. Introduction

For the magnetic fusion devices, tokamaks, plasma current control is a key requirement for their safe operation. In the present-day machines this control is based on the use of various types of inductive sensors: pick-up coils, Rogowski coils, saddle loops, etc. [1]. Despite having different engineering designs, the inductive sensors share one common physical measurement principle: the sensor signal is related to variation in the magnetic flux through the sensor loop. Time integration is necessary to retrieve the measured current. Being well established, this approach can be questioned for future magnetic fusion installations, which will operate in a steady-state regime. It is demonstrated that for discharges with a duration of several tens of minutes, the problem can be solved by using advanced integration techniques, as it was implemented at Tore-Supra [2] and KSTAR [3]. However, non-linear signal drifts in the magnetic diagnostics remain a significant challenge [4]. Moreover, during nuclear plasma operation the useful signal will be close to zero and strong radiation-induced parasitic currents will create significant perturbations. Integration of parasitic currents generated by nuclear radiation may result in a large drift error of the plasma current estimation [1].

An alternative to improving the inductive sensors is the use of proportional sensors. Currently, such options for plasma current measurements at ITER are the Hall sensors [5] and the Fibre Optics Current Sensor (FOCS) [6]. FOCS operation is based on the Faraday effect in optical fibres: light polarisation rotation as a result of interaction with external magnetic fields aligned with the fibre’s axis. It follows from the Ampere theorem that when a fibre makes a loop around a current, the total polarisation rotation angle *Θ* after passing through this loop is directly proportional to the enclosed current *I* independent on the current distribution in the loop:

*Θ* = *VI*. (1)

Here *V* is the proportionality factor called the Verdet constant. For silica fibres *V* ~ 0.7 µrad/A at 1550 nm. For tokamaks, where the currents are in an MA range, this rotation is easily detectable. Important advantages of FOCS are an easy installation, electrically passive operation, small footprint with only one fibre, and a possibility for the fibre replacement in case of a degradation or an upgrade of the system.

The ITER FOCS system will use optical fibres placed around the vacuum vessel (VV) to measure the ITER toroidal current by implementing the polarisation detection approach [7]. The ITER FOCS installation has several innovative and unique features, which were described in detail in previous publications [8,9]. According to the ITER project requirements, at maximal plasma current of 15 MA and in the absence of fast disturbances, the plasma current shall be controlled to less than ±2% or ±0.05 MA, whichever is less restrictive [10]. For the plasma current measurements, ±1% accuracy is defined [11].

The experience of using FOCS at big fusion facilities is limited with a few examples [12,13], and that experience is not completely adequate for ITER due to low radiation levels, smaller size of the devices, short-term operation, etc. As a result, the performance of the ITER FOCS cannot be predicted from the available experimental data. To bridge this gap FOCS systems were installed at JET. The primary goal of this installation was to address the system performance in a real tokamak environment, and specifically during nuclear D-T operation. Thanks to the recording, in addition to the Stokes parameters, the signal intensity, the general problem of Radiation-Induced Attenuation in optical fibres could also be addressed. JET FOCS data acquisition performed measurement with 20 ms integration intervals, which made it possible to observe transient effects and to analyse possible correlations with the neutron flux, specifically with the 14 MeV component.

The performance of FOCS during D-T operation is influenced not only by radiation but also depends on the system characteristics and interference from measurement environment. The significance of those two effects was addressed in the previous publications [14,15,16,17,18].

## 2. JET FOCS Installation

JET FOCS system relies on the use of spun fibres, specialty optical fibres produced by spinning the fibre preform during the draw process. This spinning results in rotation of the birefringence axes, making the fibre suitable for current measurements thanks to the quenching of the linear birefringence effect.

As soon as the fibre is rotated in the viscous state, spinning does not result in torsional stresses. Therefore, it can be expected that a spun and a standard fibre made from the same preform should have the same response to radiation. On the other hand, there are several reports where a significant impact of ionising radiation on the Verdet constant of the SMF-28 fibre has been observed [19,20].

**Figure 1:**
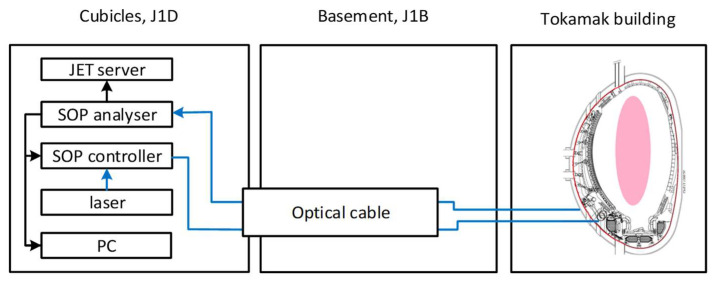
Schematics of the JET FOCS 2 installations. Laser-LS5-c-29B-20-NM, Thorlabs Inc. (Newton, NJ, USA) SOP—(state of polarisation) Controller-DPC5500, Thorlabs Inc.; SOP Analyzer—IPM5500, Thorlabs Inc.

Two FOCS systems were installed at JET. In the present case we discuss the FOCS-2 system only. Results obtained with FOCS 1 were described in the previous publications [16,17]. FOCS 2 system was installed in 2018. It was based on the radiation hard pure silica core LB1300 (Crystal Techno, Moscow, Russia) with 5 mm spin, 6.5 µm core, cut-off wavelength ~1220 nm, initial spectral losses at 1550 nm of 0.22 dB/km, and numerical aperture of ~0.15. The data acquisition modules were located in the JET J1D instrumentation area and the optical link was made using a 100 m long armoured military grade optical cable with the standard SMF-28 fibre, see Figure 1. This configuration was described in detail in the previous publications [16,17] and it corresponds well to the future ITER FOCS implementation [9]. Thanks to the use of the polarisation detection approach adopted for FOCS, the system was expected to be tolerant to the most common effect of radiation-induced transmission degradation [9]. However, the data-acquisition electronics cannot withstand the radiation levels near the VV and must be placed at a sufficient distance. In ITER optical links with a length in a range of ~100 m are inevitable.

The most important differences between FOCS 1 and FOCS 2 are the presence of the 100 m long fibre link and the use of the pure silica core sensing fibre in FOCS 2 instead of the Ge-doped core fibre in FOCS 1. Both modifications are necessary for future ITER installation but may have negative impacts on the FOCS performance. The long link can introduce polarisation perturbations. For the sensing fibre, the important parameter is the ratio of the linear polarisation beat length L_b_ to the spin period L_s_, with big values giving better performance. For installed pure silica core, the L_b_ is ~30 cm, while for Ge-doped core fibre it is in a range of several metres. It turned out that the actual performance of both systems was practically the same. It can be explained by the following reasons. The JET pulse duration is less than 80 s. During this relatively short period, changes in the polarisation properties of the link were insignificant thanks to the use of the protected fibre. For the pure silica core fibre, the ratio of L_b_/L_s_ is ~60, which is well above the limit of L_b_/L_s_ of ~20 required to achieve 1% measurement accuracy.

## 3. FOCS Radiation Environment During D-T Operation

To assess possible radiation effects on the FOCS performance, the FOCS data need to be correlated with the neutron flux at the FOCS location. The neutron fluence data can be obtained from JET KN1 Neutron flux monitor or the KN3 Neutron cameras. The KN1 signals are absolutely calibrated for measuring the neutron yield with an accuracy of 10% [21], while the signals of KN3 are not. Therefore, KN1 is the preferred option for absolute fluence measurements.

The KN1 diagnostic consists of three pairs of moderated fission chambers containing 235 U and 238 U, respectively, mounted in moderator packages at locations on the transformer magnet limbs at the torus mid-plane in three Octants, 2, 6 and 8 [22]. The absolute neutron rates are calculated from the counts recorded in the fission chambers multiplied by calibration factors determined during cross calibration of the fission chambers with the activation system (KN2). Calibration factors are specific to each detector.

Using the paired fission chambers allows one to cover the large dynamic range required for DD and D-T reactions. The U235 detector covers the range 1 × 10^10^ to 1 × 10^18^ n/s [21], and the U238 covers the range 1 × 10^16^ to 1 × 10^21^ n/s [23].

The chambers were calibrated with respect to a standardised 252 Cf fission source located inside the torus vessel in 1984/9, and that calibration has been maintained over the years by cross-calibration to the in-vessel activation system. Details on KN1 calibration are also given in [21,24].

In preparation of the DET2 campaign neutron spectra, fluxes and neutron dose rates at the position of the FOCS on the tokamak were calculated using MCNPJ [25]. The neutron spectra were calculated in 175, 640 and 709 energy group structures for further analysis. The neutron spectra for the D-T plasma in the UU model are given in [25].

According to [25] the average neutron flux at the FOCS position during D-T campaign normalised to one source neutron is 3∙10^−6^ n/cm^2^. For the FOCS position the dose rate to silicon related to neutrons was also calculated. This was achieved with the use of flux-to-dose conversion factors from ASTM E722-19 [26]. For the D-T operation the dose normalised to a source neutron is 4.13∙10^−19^ Gy. The dose rates related to gamma radiation from different sources, such as delayed gammas from activated JET components, prompt gamma rays from reactions of neutrons with materials of JET, high energy X-rays from electron bremsstrahlung and high energy gamma rays from reactions in plasma were not computed. In general, the delayed contribution is small, and the rest should be proportional to the neutron flux/fluence. The DPA in SiO_2_ in FOCS was also calculated using the methodology presented in [27]. The normalised DPA per source particle was calculated to be 2∙10^−27^ DPA/source particle.

**Figure 2:**
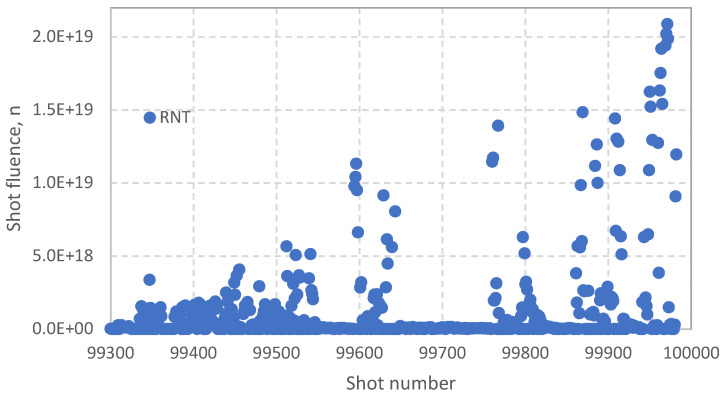
Neutron shot fluence during DTE2 operation, with the first D-T shot 99300 on 9 August 2021 and the last shot 99982 on 21 December 2021.

**Figure 3:**
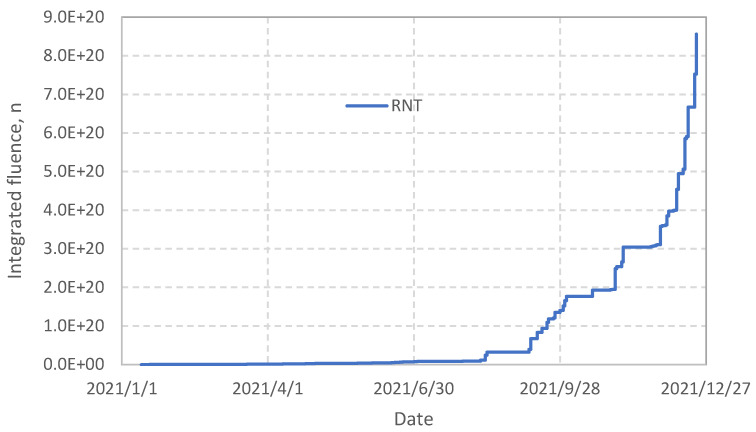
Integrated (cumulative) neutron production during 2021. DTE2 campaign started on 9 August 2021.

The actual neutronic data measured with the KN1 system during DTE2 are presented in Figure 2 and Figure 3.

The start of the DTE2 campaign was on 13 August 2021, shot #99330. It corresponds to an increase in the neuron production in Figure 3. During high power D-T shots the U235 signals saturate and the signal processing switches to the U238 Fission Chambers, seamlessly merging intershot with carefully calibrated transitions. A total D-T neutron fluence for the DTE2 was 8.5 × 10^20^ n. The JET safety case defined 2 × 10^21^ n limit on the 14 MeV neutron fluence on the VV over the JET lifetime.

## 4. FOCS Operation During DTE2

It is instructive to compare measurement results during T-T operation campaign C40 before DTE2, when the neutron fluxes were low, and D-T campaign C41, with high neutron fluxes.

One of the main goals of the JET operation was the development of main scenarios to achieve the high fusion power up to 10 MW for at least 5 s as well as good plasma confinement [28]. The choice of plasma current is important in achieving this goal. It is constrained by the need for alpha particle confinement at too low Ip and increased difficulties to control the plasma edge density and radiation with good neutron performance at higher Ip [29]. During DTE2 two main scenarios were investigated. The base-line scenario relied on high plasma currents Ip ≈ 3.5 MA and the safety factor q95 ≈ 3 (safety factor q at the 95% poloidal flux surface) [30]. The hybrid scenario was achieved at lower plasma currents Ip ≤ 2.6 MA and the safety factor q95 ≈ 4.8 [29].

Following the idea of minimising tritium consumption and neutron activation of the device during D-T operation, the plasma scenario optimisation for the D-T operation started with pure D and T as the working gas. During this phase, the choice of Ip and the magnetic field strength (BT) was made, and other challenges were also tackled: divertor heat load mitigation, control of high Z impurities, optimised H-mode entry, etc. The use of T in 2021 was an important step, because differences in physics between D and D-T are less pronounced in T-plasma as compared to D-plasma, but neutron production, which leads to activation of the JET VV, remained low. To mitigate the risk of not achieving high fusion power (target 10 MW for 5 s) both scenarios were used in the D-T operation preparation.

**Figure 4:**
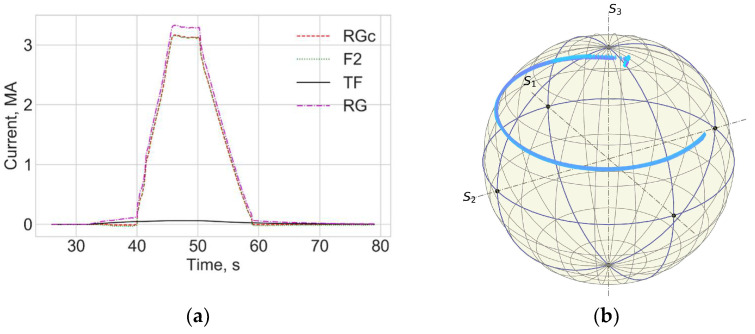
JET Shot 98655. Current profiles (**a**) and the FOCS data the trace on the rotated PS (**b**). RG—CER data, RGc—corrected CER data; TF—TF coil current; F2—FOCS-2 measurements. S_1_, S_2_, S_3_ are the components of the Stokes vector.

**Figure 5:**
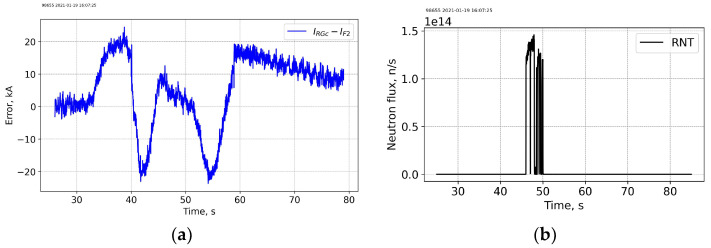
Shot 98655. Current measurement error (**a**) and the neutron fluence (**b**). RNT—total neutron flux.

Figure 4 and Figure 5 show data for FOCS 2 during start C40, preparation for T-operation, shot 98655 on 19 January 2021 16:07:25, with Ip = 3.2 MA and BT = 2.5 T. Comparison (error estimation) is made using the data for the FOCS and the Continuous External Rogowski (CER) corrected for the Toroidal Field (TF) coil current. Computation of the correction current, which is proportional to the TF current, is described in detail in [15,16]. The reason for the cross-sensitivity is non-uniformity of the cable used in CER. Making the CER cable completely uniform is a challenging technical problem, while for an optical fibre a high special uniformity over several tens of metres is easily achievable. After the correction the FOCS and CER curves appear superimposed because the difference is much smaller than the absolute value of the current, as shown in the associated error plots, e.g., Figure 5a.

**Figure 6:**
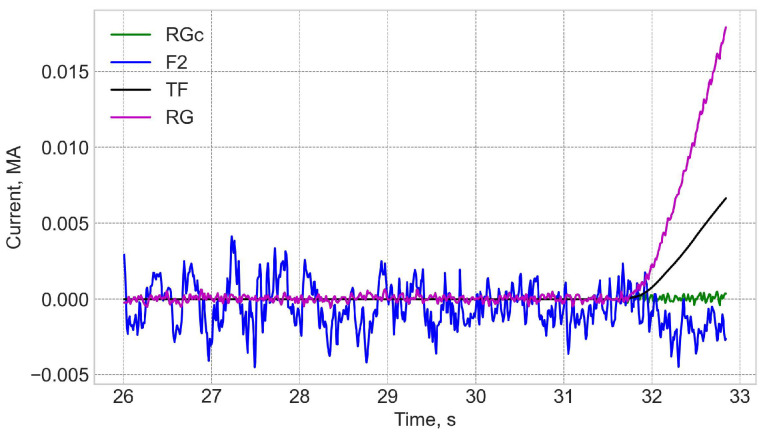
Shot 98655. Comparison of Rogowski (RG), corrected Rogowski (RGc), and FOCS (F2) measurements in the zero current operation. TF—toroidal field coil current.

It is useful to note that the intrinsic noise level of the FOCS is an order of magnitude higher than that of Rogowski, Figure 6. This difference is a consequence of an insufficient mechanical protection of the long connecting optical cable based on the standard SMF-28 type fibres, which pick up mechanical perturbations. The use of a better protection or a reflective configuration with a Faraday mirror allows reduction of this noise to the level defined by the optical hardware, which is comparable to that of Rogowski. Slow variations in the error are related to the FOCS trace deviations from the reference plane. For this shot the FOCS trace on the Poincaré sphere (PS) can be fit to a plane with S3 = 0.415 ± 0.041. The standard deviation of 0.04 corresponds to the measurement errors up to 25 kA.

The radiation level for shot 98655 is shown in Figure 5. The neutron production is related to residuals or tritium. It is negligible as compared to the subsequent D-T operation. There is no correlation between the neutron flux and the current measurement error.

**Figure 7:**
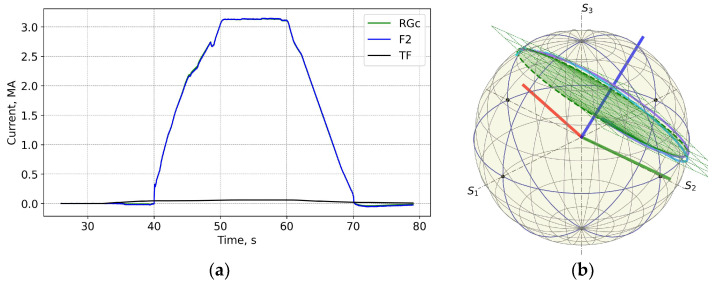
Shot 98696. Current profiles (**a**) and the measurement trace on the PS (**b**). For details see caption for the previous figures. The three coloured lines in (**b**) represent the rotated basis. S_1_, S_2_, S_3_ are the components of the Stokes vector.

**Figure 8:**
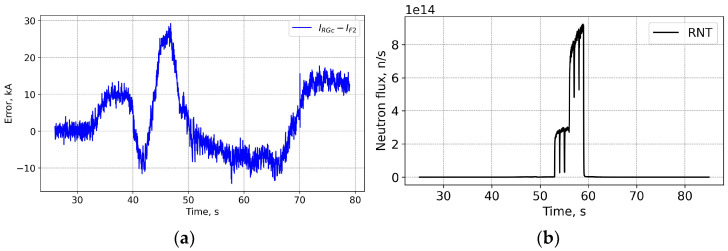
Shot 98696. Current measurement error (**a**) and the neutron fluence (**b**). RNT—total neutron flux.

The shot 98696 on 25 January 2021 18:04 is one on the first day of T-operation. Figure 7 and Figure 8 show that while the neutron fluences are ×5 times higher than for shot 98655, the measurement error trend during the shot is similar to the previously shown one. The shape of the FOCS measurements error is explained by three contributing factors. Before t = 33 s the current system is inactive and both the CER and the FOCS measure zero current. With the start of the TF coils operation a current is detected by the CER even though there is no actual current through the CER loop. The crosstalk compensation is not perfect, which results in the difference between the CER and FOCS data. At t = 40 s a fast increase in the plasma current starts. The two systems use time integration. For FOCS the integration allows the reduction of signal fluctuations, while for CER the integration is inherent for the current measurements. The time constant for FOCS is 20 ms, while for Rogowski it is ~100 ms. As a result, in both cases the measurement result is influenced by the rate of the plasma current increase. Since the integration constants (response times) are different, the scale of the influence is also different, which gives an additional contribution to the error variation over 40–50 s and 60–70 s intervals. The slow error variation over the constant plasma current interval 50–60 s is explained by the drift of the systems. For CER the main contribution comes from the integrators, while for FOCS it stems from temperature and vibrations induced birefringence variations in the connecting optical fibres. These drifts are the reason for the error at 80 s, when the current system is again inactive.

This explanation is qualitatively valid for all shots. Since the contribution of each factor is changing from one shot to another, the quantitative picture may differ significantly.

**Figure 9:**
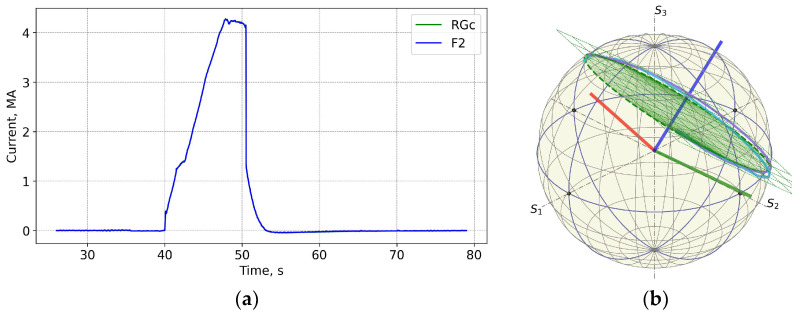
Shot 99282. Current profiles (**a**) and the measurement trace on the PS (**b**). FOCS data (F2) completely overlap the corrected Rogowski (RGc) trace. For details see caption for the previous figures.

**Figure 10:**
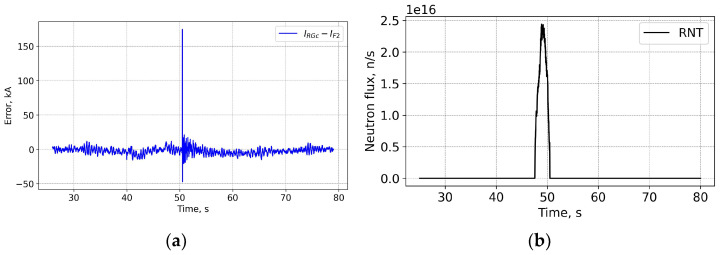
Shot 99282. Current measurement error (**a**) and the neutron fluence (**b**). RNT—total neutron flux.

Injection of hydrogen ELM pacing pellets was attempted to achieve flat top plasma operation for the entire programmed duration of ~5 s. However, at the base-line scenario 3.5 MA/3.35 T this goal could not be achieved for all T-T plasmas. Shot 99282, Figure 9 and Figure 10, is one where the T plasma sustained for the longest time. In this pulse 28 MW of NBI power and 3.5 MW of ICRH power were injected in the plasma and H pellets were injected at 25 Hz until t = 48.8 s and at 17 Hz thereafter.

In Figure 10 the spike at ~51 s is a consequence of the abrupt current quench combined with the difference in the response times of the FOCS and Rogowski. The time constant difference results in a difference in the responses, which is particularly strong at fast current changes.

**Figure 11:**
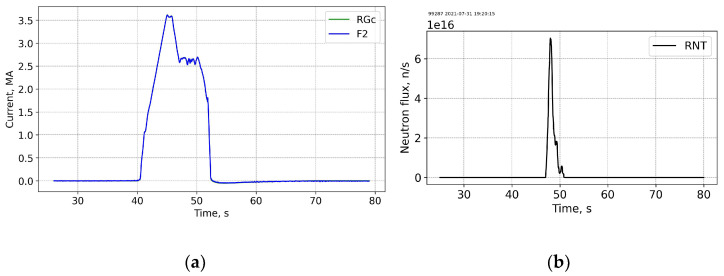
3.5 MA Shot 99287 (basic scenario), end of T-T. Current profiles (**a**) and the neutron fluences (**b**). RGc—corrected CER data, F2—FOCS-2 measurements. FOCS data (F2) completely overlap the corrected Rogowski (RGc) trace. RNT—total neutron flux.

**Figure 12:**
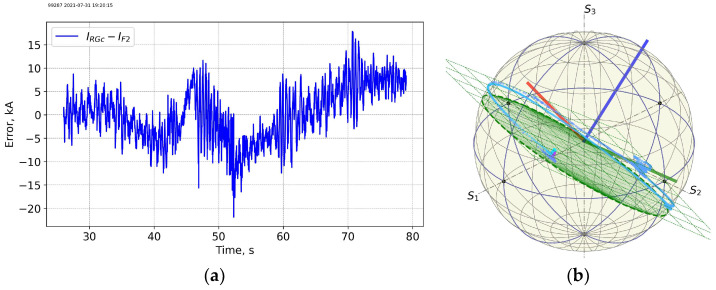
Shot 99287. Current measurement error for FOCS 2 (**a**) and the measurement trace on the PS (**b**). For details see caption for the previous figures.

Figure 11 and Figure 12 show data for another base-line scenario shot 99287 with a ~3.5 MA max Ip current (end of the T campaign) on 31 July 2021 20:20. The neutron flux is almost two orders of magnitude higher than the flux at the beginning of T-T campaign. The measurement trace on the PS is less “regular” as compared to the trace at the start of the T-T campaign.

**Figure 13:**
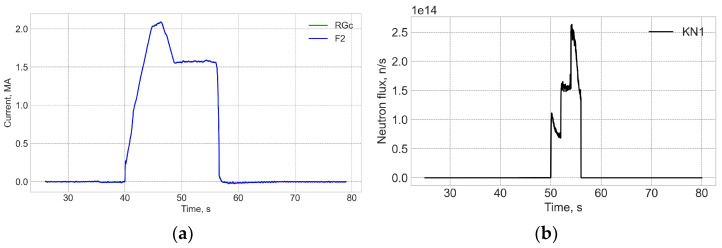
2 MA Shot 99288. Ip = 2 MA, BT = 1.4 T, end of T-T. Current profiles (**a**) and the neutron fluences (**b**). RGc—corrected CER data, F2—FOCS-2 measurements. FOCS data (F2) completely overlap the corrected Rogowski (RGc) trace. KN1—total neutron flux.

**Figure 14:**
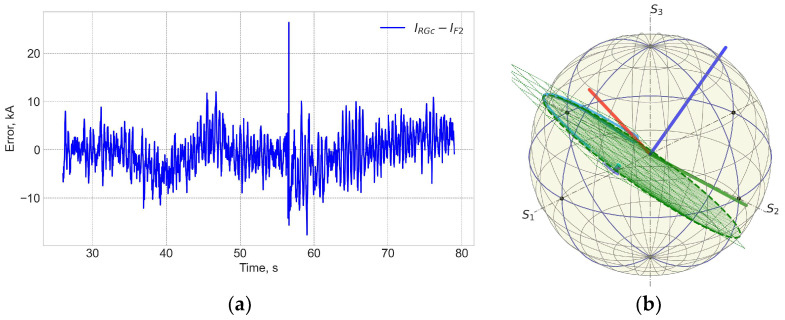
Shot 99288. Current measurement error for FOCS 2 (**a**) and the measurement trace on the PS (**b**). For details see caption for the previous figures.

The high current operation scenario of shots 99282 and 99287 can be compared with that for the shot 99288, with the max Ip ~2 MA, as shown in Figure 13 and Figure 14. In the latter case the neutron flux is more than two orders of magnitude lower as compared to the shot 99287. The difference with the Rogowski coil is also lower. The spike at ~57 s is again a consequence of the difference in the response times of the FOCS and Rogowski.

Even though it was not possible to achieve a stationary plasma at 3.5 MA/3.35 T base-line conditions for the 5 s time requested for the D-T operation, it was decided to use the base-line scenario in D-T. It was expected that injecting ELM pacing pellets at a higher frequency, the availability of a higher NBI power coming from the D beams, and a particle confinement lower in D-T as compared to T plasma would allow the compound ELM regime to be maintained and to achieve a stationary discharge for the target 5 s duration. A 2.3 MA current was maintained for the hybrid scenario.

**Figure 15:**
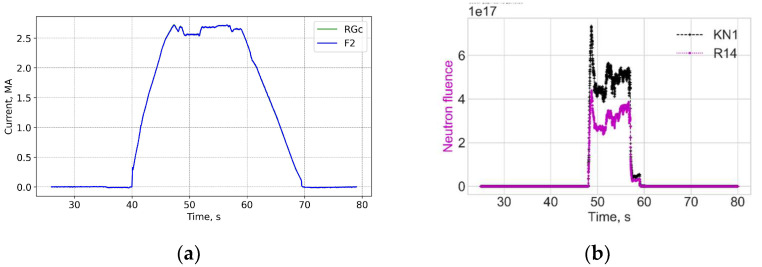
Shot 99347. Current profiles (**a**) and the neutron fluences (**b**). RGc—corrected Rogowski data; F2—FOCS-2 measurements. FOCS data (F2) completely overlap the corrected Rogowski (RGc) trace. KN1—total neutron fluence, R14—fast neutrons.

**Figure 16:**
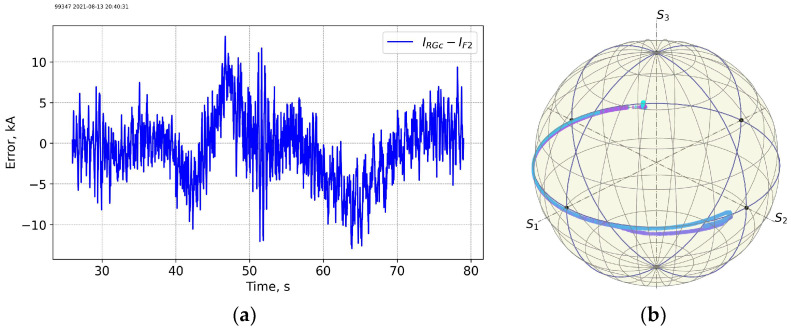
Shot 99347. The measurement error (**a**) and measurement trace on the PS (**b**).

Figure 15 and Figure 16 show data for shot 99347 at the start of the D-T operation on 13 August 2021 with 18 MW heating and 50/50 D-T mixture in the hybrid scenario. The neutron flux is an order of magnitude higher as compared to the T-T operation. However, the variation in the measurement error for FOCS 2 during the shot looks like that before the start of the D-T campaign.

**Figure 17:**
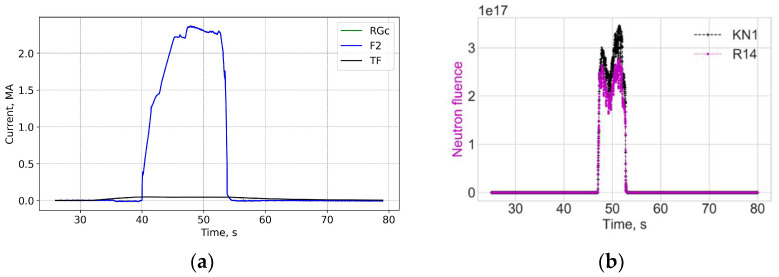
Shot 99491. Current profiles (**a**) and the neutron fluences (**b**). RGc—corrected Rogowski data; F2—FOCS-2 measurements. FOCS data (F2) completely overlap the corrected Rogowski (RGc) trace. TF—TF coil current. KN1—total neutron fluence, R14—D-T neutrons.

**Figure 18:**
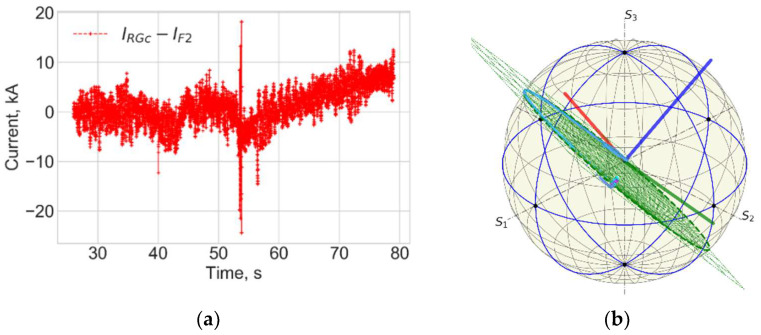
Shot 99491. The measurement error (**a**) and measurement trace on the PS (**b**). For details see caption for the previous figures.

Figure 17 and Figure 18 show data for another 50/50 D-T mixture shot 99491 on 25 September 2021 19:50:43. No significant difference with the data for the shot at the beginning of the D-T operation could be seen. The sharp peak in the errors for FOCS 2 at ~53 s is related the abrupt current decrease.

**Figure 19:**
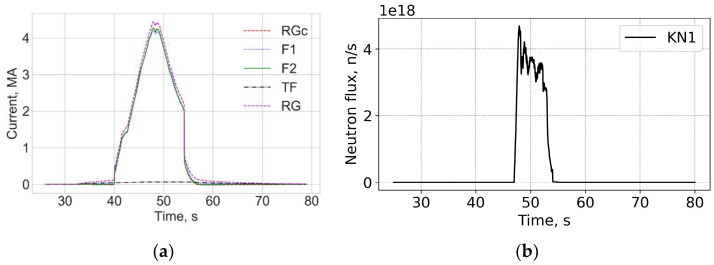
Shot 99971. Current profiles (**a**) and the neutron fluences (**b**). RG—CER data, RGc—corrected Rogowski data; TF—TF coil current; F1 and F2—FOCS 1 and FOCS 2 measurements. KN1—total neutron flux.

**Figure 20:**
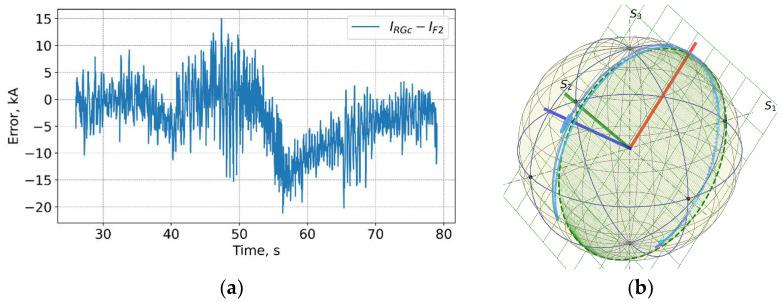
Shot 99971. The measurement error (**a**) and measurement trace on the PS (**b**).

Figure 19 and Figure 20 show data measured at the end of the D-T campaign, shot 99971 on 21 December 2021 13:23 with a record 59 MJ produced fusion power. A high peak neutron flux above 4 × 10^18^ n/s was achieved. In general, the trace on the PS for this shot is similar to other shots during DTE2 with no obvious correlation between the neutron flux and the current measurement error visible.

**Figure 21:**
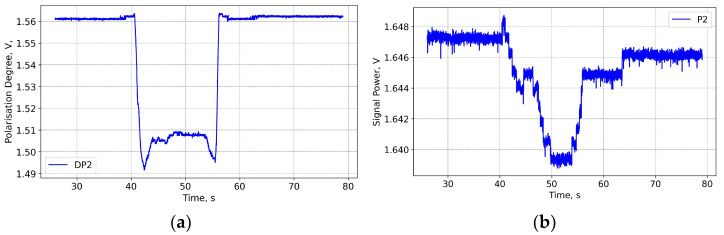
Shot 99971. Polarisation degree (**a**) signal amplitude (**b**).

FOCS operation is based on the polarisation rotation detection. Although the Stokes vector is normalised and the polarisation rotation detection does not depend on the degree of polarisation (DOP), it is useful to check how DOP can be modified. In Figure 21 the DOP is represented for the Shot 99971 in arbitrary (V) units. According to the IPM5300 specification, the DOP signal is from −2.5 to + 2.5 V. +1.5 V signal corresponds to 100% polarised light, 0 V to 62.5%. In the present case the maximal signal is +1.56 V, which formally corresponds to a DOP of 101.5%. In practice, the value of +1.56 V should correspond to nearly 100% polarised light, but because formally it corresponds to more than 100% DOP we are not able to provide the exact DOP conversion. The conclusion here is that there is a small but detectable decrease in the polarisation degree, which can be correlated with start and the end of plasma operation.

Depolarisation is a common effect when a polarised light propagates in a SMF. It can originate from averaging either spatial, for example, as a result of polarisation or chromatic dispersion [31], or temporal, because of instability of fibre polarisation properties. For a spun fibre the depolarisation length due to the dispersion effect is estimated as follows [32]:

$$L_{D}=\frac{\lambda }{\Delta \lambda }L_{b}^{2}{\left(\frac{1}{4L_{b}^{2}}+\frac{1}{L_{s}^{2}}\right)}^{1/2}$$

where *λ* is the source wavelength, Δ*λ* is the source spectral width, *L_b_* is the polarisation beat length and *L_s_* is the spin period. In the case of Lo-Bi spun fibre used in JET, *L_D_* > km and this effect can be safely neglected.

It is also possible that polarisation degradation is a result of light injection via the radioluminescence effect. There are two possible origins for radioluminescence in optical fibres: optical transitions in radiation-induced defects and Cherenkov radiation. For the former, the radioluminescence spectrum depends on the fibre core chemical composition. In the present case only pure silica core fibres need to be considered. The Ge-doped link fibres are located far from the vacuum vessel and are subject to very low levels of radiation. Radioluminescence peaks in rad-hard fibres are in the visible range, at 450, 460 and 650 nm [33]. These wavelengths are outside the FOCS wavelength operation range and are not well transmitted through Ge-doped SMF-28 type fibres. Cherenkov emission has a broad spectrum, with the maximum in the UV range, which can also extend to the infrared. If that be the case, we could expect that the signal intensity grows in correlation with the polarisation degradation. However, Figure 21 shows a small decrease in the signal amplitude, and there is no direct correlation between the degree of polarisation and the amplitude variations. We can conclude that time dependent perturbations of the FOCS signal are the most likely reason for the observed polarisation degradation. At this moment it is not clear how this effect could be used to improve the FOCS performance.

**Figure 22:**
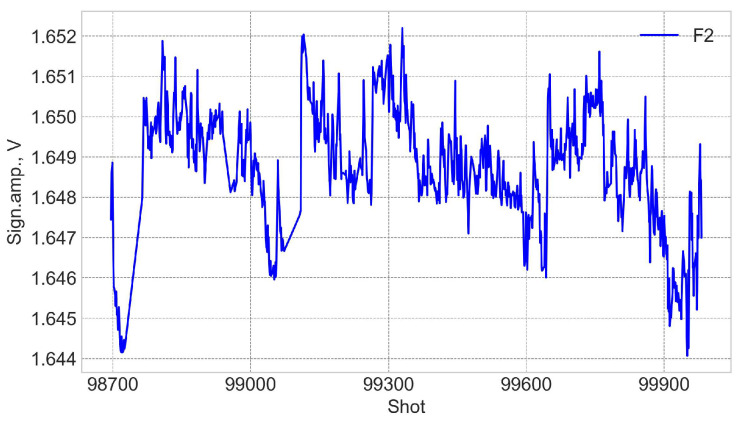
Variations in the optical signal power during 2021. Start D-T at shot 99331.

Variations in the FOCSs signal amplitude during 2021 are shown in Figure 22. The signal amplitude is represented by a voltage in a range from −2.5 to 2.5 V, corresponding to the power on the logarithmic scale from −30 to + 20 dBm. Thus, the signal of 0 V corresponds to −5 dBm. The stability of the laser source (LS5-c-29B-20-NM, Thorlabs Inc.) is better than ±0.01 dB in 24 h. This value corresponds to ±1 mV or ±0.06% variations of the signal amplitude, which is an indication that the short-term variations are due to the source power variations. On the long term the signal variations are only ×3 bigger (within ±0.2%) and could also be attributed to the source power variations. From the point of view of nuclear operation, it is important that no obvious degradation trend, which could be directly correlated with the neutron accumulation, is observed during D-T operation.

The systematic measurement error can grow as a result of neutron radiation induced change in the Verdet constant. A significant impact of gamma radiation on the Verdet constant of the standard SMF-28 fibre has been observed [19,20]. To analyse such a possible effect, we consider the Verdet constant as a parameter, which can be adjusted to obtain a best match between the FOCS and the continuous Rogowski. Before a shot, variations in the reference external Rogowski system are within ±0.5 kA, while variations in FOCS 2 are in a range of ±5 kA. FOCS fluctuations are related to the accuracy of the Stokes parameter measurements and perturbations of the sensing and the link fibres and are not related to possible Verdet’s constant changes. To find the Verdet constant we used data for currents not smaller than 50% of the maximal current during the shot and averaged zero current data.

**Figure 23:**
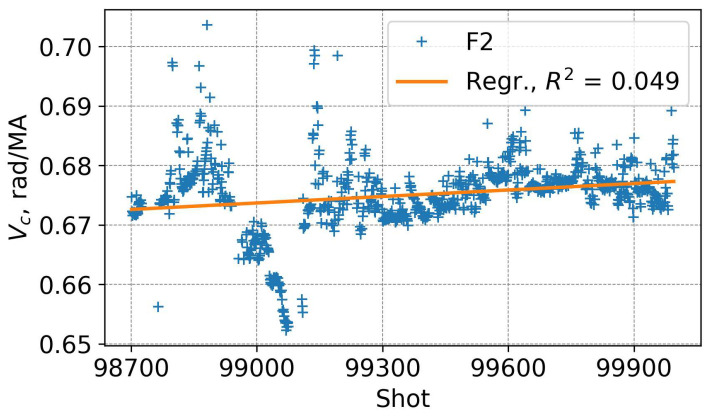
Variation in the normalised Verdet constant for FOCS 2 during 2021 operation. Start D-T at shot 99331.

Values of the Verdet constant obtained in this way are shown in Figure 23. Shot-to-shot variations up to ±4% are observed. When using a linear fit to the data, an increase of ~0.7% can be guessed for FOCS 2 during 2021 operation. However, a small R2 value indicates that the mean value should provide a better estimation than the linear function. And it is not possible to conclude if this trend is physically present or not. A more detailed inspection of the outliers shows that in those cases the measurements were not accurate, for example, due to a relatively low maximal currents combined with rapid variations or a short pulse duration.

## 5. Conclusions

To operate a tokamak in an optimal way and achieve a maximum fusion power, the plasma current must be optimised. The choice of plasma current is constrained by various factors such as the need for alpha particle confinement, avoiding hot spots in the machine at low Ip, and increased difficulties to control the plasma edge density and radiation with good neutron performance at higher Ip. To achieve this goal the plasma current measurements system must always be available and satisfy the performance requirements. The tokamak harsh environment makes this task quite challenging.

Fibre Optics Current Sensor (FOCS) will be used at ITER to perform plasma current measurement during quasi-steady state D-T plasma operation. This system is classified as SIC-2 and the reliability of its operation must be demonstrated. Effects of the tokamak harsh environment on the FOCS performance must be evaluated to predict possible failure modes and relevant mitigation measures. The influence of nuclear radiation with the significant flux of 14 MeV neutrons is of specific concern. This problem was addressed by operating the FOCS during D-T campaigns at JET.

Detailed analysis of the JET radiation environment during nuclear D-T operation has been presented in [34]. During DTE2 campaign ~0.85 × 10^21^ neutrons were produced. This is factor ×10^6^ smaller than the expected neutron production during ITER lifetime. Thus, JET FOCS results may look to be not relevant for ITER. However, when the peak neutron yield rates are considered, DTE2 values of (1–3) × 10^18^ n/s were regularly obtained with a peak rate of 4.5 × 10^18^ n/s for the record shot 99971. In ITER it is expected to reach 1.8 × 10^20^ n/s for a 500 MW pulse. This means that the neutron yield rate ratio between ITER and JET is ~35 [34]. Moreover, JET VV is smaller than the ITER VV and the VV thickness is also smaller. In ITER the estimated neutron flux for FOCS is from 3.8 × 10^8^ to 7.7 × 10^10^ n/cm^2^/s [35].The neutron flux to be considered for the ITER FOCS design is 9 × 10^10^ n/cm^2^/s [36]. According to [25] the average neutron flux at the FOCS position during D-T operation is defined with the normalisation constant of 3∙10 ^6^ n/cm^2^ to one source neutron. Therefore, the peak rate during DET2 corresponds to 1.5 × 10^12^ n/cm^2^/s at the FOCS location, i.e., more than an order of magnitude higher than the design value for the ITER FOCS. This means that we can consider that JET results are representative for FOCS operation at ITER relevant flux for a duration of ~300 s.

In terms of transient effects, the JET environment during D-T operation is more severe than the design conditions for the ITER FOCS, and the presented results provide a confirmation that ITER FOCS will reliably measure the plasma current during the initial phase of the D-T operation. It is possible that extended ITER D-T operation will influence the FOCS operation, but the presented results indicate that such degradation will be sufficiently slow.

There is also a possibility to extend FOCS operation lifetime by using fibre transmission recovery technique. Temperature annealing or radiation-induced defects will naturally occur during ITER vacuum vessel baking interval. Another option is to use photobleaching. It allows a fibre recovery practically at any time and deserves to be investigated.

## Data Availability

Original data are available on the JET data server.

## Abbreviations

The following abbreviations are used in this manuscript:

JET	The Joint European Torus
FOCS	Fiber Optics Current Sensor
VV	Vacuum Vessel
